# Hospital-Based Health Professionals’ Perceptions of Frailty in Older People

**DOI:** 10.1093/geront/gnae041

**Published:** 2024-05-07

**Authors:** Kisani Manuel, Maria Crotty, Susan E Kurrle, Ian D Cameron, Rachel Lane, Keri Lockwood, Heather Block, Catherine Sherrington, Dimity Pond, Tuan A Nguyen, Kate Laver

**Affiliations:** Division of Rehabilitation, Aged and Palliative Care Service, Southern Adelaide Local Health Network, Bedford Park, South Australia, Australia; Department of Rehabilitation and Aged Care, College of Medicine and Public Health, Flinders University, Bedford Park, South Australia, Australia; Division of Rehabilitation, Aged and Palliative Care Service, Southern Adelaide Local Health Network, Bedford Park, South Australia, Australia; Flinders Health and Medical Research Institute, College of Medicine and Public Health, Flinders University, Bedford Park, South Australia, Australia; Faculty of Medicine and Health, University of Sydney, Sydney, New South Wales, Australia; Department of Rehabilitation and Aged Care Services, Northern Sydney Local Health District, Hornsby, New South Wales, Australia; Department of Rehabilitation and Aged Care Services, Northern Sydney Local Health District, Hornsby, New South Wales, Australia; John Walsh Centre for Rehabilitation Research, Faculty of Medicine and Health, University of Sydney, Sydney, New South Wales, Australia; Flinders Health and Medical Research Institute, College of Medicine and Public Health, Flinders University, Bedford Park, South Australia, Australia; Faculty of Medicine and Health, University of Sydney, Sydney, New South Wales, Australia; Caring Futures Institute, College of Nursing and Health Sciences, Caring Futures Institute, Flinders University, Bedford Park, South Australia, Australia; Faculty of Medicine and Health, University of Sydney, Sydney, New South Wales, Australia; Sydney Musculoskeletal Health, Institute for Musculoskeletal Health, University of Sydney and Sydney Local Health District, Sydney, New South Wales, Australia; Wicking Dementia Research and Education Centre, University of Tasmania, Hobart, Tasmania, Australia; Social Gerontology Division, National Ageing Research Institute, Melbourne, Victoria, Australia; Department of Psychological Sciences, School of Health Sciences, Swinburne University of Technology, Melbourne, Victoria, Australia; Division of Rehabilitation, Aged and Palliative Care Service, Southern Adelaide Local Health Network, Bedford Park, South Australia, Australia; Caring Futures Institute, College of Nursing and Health Sciences, Caring Futures Institute, Flinders University, Bedford Park, South Australia, Australia

**Keywords:** Communication, Guidelines, Hospital, Implementation barriers, Qualitative

## Abstract

**Background and Objectives:**

There is a high prevalence of frailty amongst older patients in hospital settings. Frailty guidelines exist but implementation to date has been challenging. Understanding health professional attitudes, knowledge, and beliefs about frailty is critical in understanding barriers and enablers to guideline implementation, and the aim of this study was to understand these in rehabilitation multidisciplinary teams in hospital settings.

**Research Design and Methods:**

Twenty-three semistructured interviews were conducted with health professionals working in multidisciplinary teams on geriatric and rehabilitation wards in Adelaide and Sydney, Australia. Interviews were audio recorded, transcribed, and coded by 2 researchers. A codebook was created and interviews were recoded and applied to the Framework Method of thematic analysis.

**Results:**

Three domains were developed: diagnosing frailty, communicating about frailty, and managing frailty. Within these domains, 8 themes were identified: (1) diagnosing frailty has questionable benefits, (2) clinicians don’t use frailty screening tools, (3) frailty can be diagnosed on appearance and history, (4) frailty has a stigma, (5) clinicians don’t use the word “frail” with patients, (6) frailty isn’t always reversible, (7) there is a lack of continuity of care after acute admission, and (8) the community setting lacks resources.

**Discussion and Implications:**

Implementation of frailty guidelines will remain challenging while staff avoid using the term “frail,” don’t perceive benefit of using screening tools, and focus on the individual aspects of frailty rather than the syndrome holistically. Clinical champions and education about frailty identification, reversibility, management, and communication techniques may improve the implementation of frailty guidelines in hospitals.

## Background and Objectives

Frailty is an increasing issue for the Australian population. Current estimates suggest that 21% of Australians aged 65 years or older are frail and another 48% are prefrail (Thompson et al., 2018). By 2050, it is predicted that 4 million Australians aged 70 years or older will either be frail or at-risk of frailty (Blyth et al., 2008). Frailty is described as a dynamic state of heightened vulnerability to stressors (Fried et al., 2021) and people with frailty are at higher risk of adverse outcomes, including falls, delirium, disability, and death compared to age-matched individuals without frailty (Dresden et al., 2019).

Hospital settings have a high prevalence of frailty within their patient population. Richards et al. (2019) reported that almost half (48.8%) of adult inpatients were classified as frail and prior studies reported a wide range of frailty prevalence rates of between 27% and 80% in hospitalized older patients (Andela et al., 2010; Joosten et al., 2014). Frailty is not routinely and consistently assessed in most primary, secondary, or tertiary healthcare settings. Suggested reasons for this include time and resource limitations (Ruiz et al., 2020), uncertain benefits of frailty case finding (Abbasi et al., 2018), and a lack of education and training about frailty (Taylor et al., 2017).

Hospital admissions may offer an opportunity to identify and intervene to reduce frailty. Current clinical practice guidelines exist and recommend (i) using validated tools to identify frailty, (ii) exercise incorporating resistance training, (iii) polypharmacy review, (iv) screening for reversible causes of fatigue, (v) screening and considering protein and caloric supplementation/fortification, and (vi) vitamin D supplementation if vitamin D is deficient (Dent et al., 2017).

The implementation of guidelines into clinical practice is often challenging due to the complexity and dynamic nature of the health system in which there is a rich interplay of stakeholder and contextual factors (Correa et al., 2020; Livet et al., 2018). Health professional knowledge, attitudes, and beliefs are key aspects of successful implementation of guidelines (Michie et al., 2005, 2014). For this reason, this study was conducted to explore the perspectives of health professionals in identifying, communicating, and managing frailty in inpatient settings. Health professionals’ perspectives can help us to understand contextual factors and implementation barriers for adopting clinical guidelines in practice.

Several existing studies have explored the perceptions of health professionals about frailty, but most have focused on primary care settings. These studies have revealed that frailty is generally perceived as a cycle of decline, with management focused on stabilizing frailty and preventing further decline (Ambagtsheer et al., 2019). Another common finding is that health professionals believe they can identify frailty on appearance, rendering formal screening unnecessary, and impacting on the uptake of validated screening tools (Ambagtsheer et al., 2019; Archibald et al., 2020; Lang et al., 2009). Furthermore, communication between health professionals and older people is influenced by the knowledge, capability, and attitudes held by health professionals (Lawless et al., 2020).

There is less research into health professional knowledge and beliefs in hospital settings, including in geriatric care teams, where assumed knowledge is high. Two studies involving surgical teams in hospitals identified limitations in knowledge and understanding of frailty, which were key barriers to frailty assessment and management (Archibald et al., 2020; Eamer et al., 2017). Furthermore, different meanings and interpretations of frailty amongst health professionals and with patients affect identification, management, and communication about frailty (Archibald et al., 2020).

This study aims to identify (1) perceptions and knowledge of frailty; (2) communication practices on the topic of frailty; and (3) reported clinical management of patients with or suspected of frailty.

## Research Design and Methods

### Design

Qualitative research study involving face-to-face and telephone semistructured interviews was conducted with inpatient professionals from the Southern Adelaide Local Health Network and Northern Sydney Local Health District. Ethical approval was granted as part of the FORTRESS (Frailty in Older People: Rehabilitation, Treatment, Research Examining Separate Settings) study by Northern Sydney Local Health District Human Research Ethics Committees reference number 2020/ETH01057. The consolidated criteria for reporting qualitative research (COREQ) checklist (Tong et al., 2007) was used to ensure accurate and rigorous reporting of this study (Supplementary Material, Section A).

### Participants and Setting

Participants were health professionals working in a hospital setting on six separate wards across Southern Adelaide, South Australia, and Northern Sydney, New South Wales. The included wards were a mix of acute and subacute wards. In Southern Adelaide, the three wards were located on three separate sites (all metropolitan hospitals in the same health network): a subacute geriatric evaluation and management ward in a major teaching hospital, a mixed ward of acute general medicine and subacute geriatric evaluation and management ward in a smaller metropolitan hospital, and a subacute rehabilitation ward in a smaller metropolitan hospital. The three wards in Northern Sydney were an acute cardiac and respiratory ward, a subacute rehabilitation ward, and an acute neurology and general medicine ward. They were all located at a major metropolitan teaching hospital. Both included sites have inpatient geriatric and rehabilitation services and methods of screening for frailty in different patient subgroups (ward or clinical service dependent); however, neither have widespread systematic targeted frailty identification and management throughout their health service.

A total of 24 health professionals participated in the interviews. Participants were purposefully recruited at each site. Purposive sampling is commonly used in qualitative research and involves identifying and selecting individuals or groups of individuals that are especially knowledgeable about and experienced with a phenomenon of interest (Creswell & Clark, 2017; Palinkas et al., 2015). Eligible participants were identified by the clinical leads of unit on each ward. Potential participants were invited in person to participate by a member of the research team. Health professionals were selected if they were from different specialties; medical, nursing, physiotherapists, occupational therapists, social workers, pharmacists, and allied health assistants with experiences of working with hospitalized frail older people. Written consent was obtained from participants and each interview was audio recorded. Participants were educated on the reasons for the research during the informed consent process.

### Data Collection

Interviews were facilitated by K. Manuel, K. Lockwood, or RL. K. Manuel is a rehabilitation physician who conducted all the interviews with participants in Southern Adelaide, and four of the Northern Sydney interviews. There were no significant relationships with participants prior. K. Lockwood and RL conducted the remaining interviews with participants in Sydney. K. Lockwood is a Registered Nurse and the NSW Project Coordinator for the FORTRESS Study. RL is an exercise physiologist and implementation clinician for the FORTRESS project in NSW. Neither K. Lockwood nor RL personally knew any of the participants they interviewed. The NSW interviews were audio recorded and transcribed in NSW and all audio and written data were sent to South Australia for collation and analysis.

**Figure 1.**
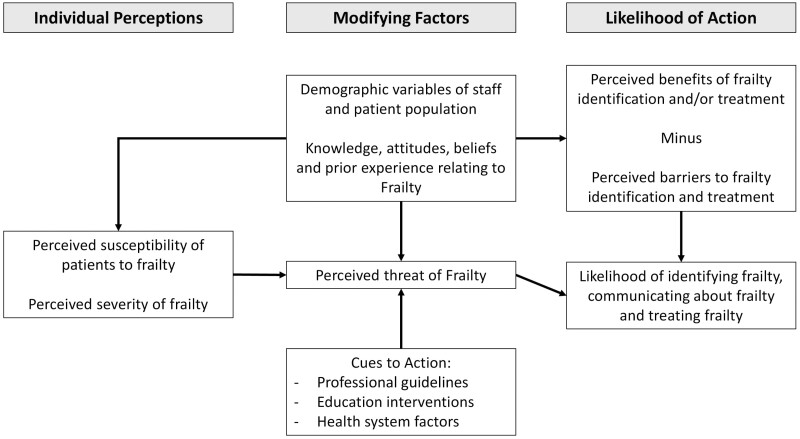
Modified health belief model used to develop interview guide, adapted from Janz and Baker (1984).

The development of the interview guide was informed by the Health Belief Model, which is a framework used to understand beliefs that influence health-related behaviors and actions (see Figure 1; Champion & Skinner, 2008). The guide was reviewed and refined by members of the research team. The interview guide included questions about frailty knowledge and perceptions, management, screening, and resources (Supplementary Material, Section B). Written consent was obtained from participants and each interview was audio recorded. Thematic saturation was monitored throughout recruitment. After 12 interviews in Southern Adelaide, no new themes were generated. An additional two interviews were conducted to confirm saturation. Interviews were then conducted in Northern Sydney. A total of nine interviews were conducted. No new themes were generated and there was agreement between K. Manuel, K. Laver, and R. Lane that thematic saturation had been reached.

### Data Analysis

Data analysis was informed by the Framework Method of thematic analysis (Gale et al., 2013). The Framework Method was developed by social researchers in the UK (Ritchie et al., 2013) and requires a systematic approach to qualitative data analysis with seven stages (see Table 1). The Framework Method is useful for the thematic analysis of qualitative interview data as it allows comparison and contrast of themes among many cases while also allowing each perspective to retain their context within each individual interview (Gale et al., 2013). It not only allows for greater transparency of the data but also allows researchers to develop a matrix to provide structure to the systematic development of codes and themes (Collaço et al., 2021).

**Table 1.** Procedure for Framework Analysis, Adapted From Gale et al. (2013)

Stage of analysis	Description
1. Transcription	Data transcribed verbatim
2. Familiarization with the interview	Data read multiple times for familiarization
3. Coding	Transcripts read and coded using Nvivo software by two researchers
4. Development of a working analytical framework/codebook	Codes compared and reviewed by three researchers. Codes grouped into categories to develop a working analytical framework
5. Applying the analytical framework	Analytical framework applied by indexing transcripts using existing codes and categories
6. Charting data into the framework matrix	Spreadsheet developed using the analytical framework to generate a matrix and data charted into the matrix
7. Interpreting the data	Data characteristics and concepts explored

Each transcript was coded by two researchers (K. Manuel and R. Lane or K. Laver) using NVivo software. Codes were developed initially based on interview content. Codes were agreed upon, collated, and arranged into a codebook by three researchers (K. Manuel, R. Lane, and K. Laver) through discussion and cross-checking coding between researchers. Interviews were then recoded using the analytical framework provided by the agreed codebook. Coded data were collated in themes and subthemes, which were further refined and confirmed by members of the research team. A narrative summary of each theme and subtheme was presented summarizing the main points and identifying linkages between themes.

## Results

A total of 23 interviews were conducted, which included 24 health professionals across six wards (geriatric and rehabilitation) in Southern Adelaide and Northern Sydney between May 2021 and September 2022. Interviews were conducted either in person (17 interviews) or over the phone (6 interviews) at a time that suited the participant availability within clinical workloads. Phone interviews were completed for two participants due to heightened COVID restrictions at the time of the interview, and the other four participants were in a different state to the interviewer. One interview included two health professionals (due to participant request). The duration of the interviews was between 10 and 31 min.

Interviewees included allied health, nursing, and medical staff working on the inpatient wards (see Table 2). All interviews were conducted at the workplace, and all participants were interviewed at their workplace except one who participated in a phone interview from home. Most interviews were conducted in private offices, but some were in shared offices (with potential for nonparticipants to be present). There were no staff members who refused to participate and no participants withdrew consent following the interview.

**Table 2.** Participant Characteristics (*N* = 24)

Variable	*n*	%
Sex		
Male	4	16.7
Female	20	83.3
Location		
Adelaide	15	62.5
Sydney	9	37.5
Health profession/role		
Physiotherapist	8	33.3
Pharmacist	4	16.7
Nurse	3	12.5
Dietitian	3	12.5
Doctor	2	8.3
Occupational therapist	2	8.3
Social worker	1	4.2
Allied health assistant	1	4.2

Thirty-six codes were identified in nine categories and agreed upon by K. Manuel, R. Lane, and K. Laver. The framework analysis process resulted in identification of three overarching domains: diagnosing frailty; communicating about frailty; and managing frailty. These domains were reflective of the aims of the study and questions in the interview guide. Eight themes within these domains were identified (see Table 3).

**Table 3.** Identified Domains and Themes

Domain	Theme
Diagnosing frailty	Diagnosing frailty has questionable benefits
	Clinicians don’t use frailty screening tools
	Frailty can be diagnosed on appearance and history
Communicating about frailty	Frailty has a stigma
	Clinicians don’t use the word “frail” with patients
Managing frailty	Frailty isn’t always reversible
	Lack of continuity of care after acute admission
	The community lacks resources

### Domain 1: Diagnosing Frailty

All participants demonstrated some knowledge about frailty and common attributes of frailty. Frail people were described as being of older age, vulnerable, having multiple comorbidities, having reduced function, appearing thin, moving slowly, experiencing falls (or near misses), displaying hunched posture, and presenting with cognitive impairment. Three health professionals described frailty as being a process of decline involving a “vicious cycle” or a “spiral of deconditioning.” Only one health professional described it as an “umbrella” disorder; however, multiple staff discussed frailty as such, comprising multiple components including physical, emotional, and cognitive impairment contributing to the condition. Most staff referred to frailty as being on a scale, or a continuum, with patients being less or more affected by frailty. Participant 8 (Occupational Therapist) stated “I consider it a scale pretty-much and every single patient who comes through here is frail to an extent.”

The majority of participants felt that they could identify patients with frailty from their appearance, “you can sort of tell … just by looking at someone, whether or not they’re frail” (Participant 9: Physiotherapist). Some participants also said that they used information from the patient’s history and collateral information from friends or family to determine if a patient was frail, particularly if the person was becoming more reliant on other people in the community and less independent.

Many participants felt that diagnosing frailty was not helpful, with Participant 11 (Occupational Therapist) stating, “I don’t think, to us, having a red flag saying someone’s frail would make a difference.”

The lack of need for a diagnosis of frailty was evident for a few reasons. Some health professionals felt that most patients in their inpatient unit were frail, so it didn’t help to distinguish frail patients from other patients or determine required interventions. Participant 12 (Nurse) mentioned that “I don’t know if they’re [frailty assessments] going to make much difference, because if you asked me if I had a look around I think most of the percentage of our patient is under the frailty criteria.”

Often participants referred to their role in the multidisciplinary team, which was specific to physical function (physiotherapy) or diet (dietitian), for example, and felt that frailty was too broad a term. They felt it didn’t help to identify the individual’s specific components of frailty that could be managed by each member of the team, with Participant 6 (Physiotherapist) describing that frailty diagnosis “can make it a bit confusing as to what aspects of frailty are important for that person’s overall picture of frailty. So I find that kind of unhelpful in some ways.”

Some participants felt that identifying frailty was useful. However, those who felt it was useful clarified that it is beneficial if it leads to changes in clinical management, such as through leading to more detailed assessment or targeted intervention.

The health professionals interviewed did not use frailty screening tools routinely in their clinical practice and many identified a lack of knowledge of validated screening tools and how to administer them. Most participants identified their own discipline-specific screening tools they use, for example, the Malnutrition Universal Screening Tool (MUST) and felt that these tools were more beneficial in identifying patients on their unit in need of input from their discipline during the admission.

### Domain 2: Communicating About Frailty

Health professionals generally avoided using the word “frail” with patients. Most participants agreed that there was a stigma associated with frailty, and that frailty sounds like a subjective label with negative connotations. Many health professionals felt that they might offend patients if they used the word “frail.”

There are a lot of losses with ageing. And you know, gosh, so many things. To be told that “you’re too frail” I think that’s a smack in the mouth actually for an elderly person. I would never say that to anybody. I say deconditioned … (Participant 7: Social Worker)

Some participants said that patients sometimes use the word “frail” to describe themselves. When this occurred, health professionals felt more comfortable using the word with the patient.

Health professionals reported that they may use the word “frail” when talking with other clinicians, as they felt there was a mutual understanding of what the term means. They also used the word “frail” occasionally with family members of patients, as a way of explaining the aging process and discharge planning needs.

If you explain frailty as a condition that happens to many people as they age, it becomes less of something that they’ve personally been responsible for. And I think that helps in the way that you then sell what you want to do. (Participant 6: Physiotherapist)

Health professionals felt that discussions about frailty need to be individualized and goal-oriented. Mostly they believed the term “frail” did not need to be used, rather individual components, such as weight loss or reduced strength, were specified. Many participants reported that the term “deconditioned” was a term that replaced “frail” in conversations with patients.

So I would never just say you’re a… generalized frail person. I would say, “Your arms are weaker, so you can’t do this,” or, “That’s causing you to be limited in this area.” We talk about tasks, I think, more than being frail or specific body function. (Participant 11: Occupational Therapist)

I think you’re always discussing frailty but in a roundabout way by talking about the consequences of frailty, like you’re at high risk of falls and things like that. You might not necessarily directly address frailty, but I think the gist of the conversation is really, it’s about frailty. (Participant 1: Medical Practitioner)

### Domain 3: Managing Frailty

Generally, participants believed that frailty could be managed and improved, but not always reversed. They reported that early intervention was important, and that successful treatment of frailty was more likely to be possible if patients were identified when they were prefrail or only mildly frail. They identified their role in the hospital setting as being limited by short hospital stay length, as well as acute issues being more highly prioritized than the patient’s frailty.

We’re not going to reverse frailty in an acute setting. (Participant 11: Occupational Therapist)

… all we’re thinking of every single day is discharge planning, how am I going to get this person out. And we do … But I don’t think that the intervention that we provide in acute is probably enough to reverse the frailty process. (Participant 9: Physiotherapist)

Like sometimes it feels like the intervention we’re providing, we’re doing just because you’re sort of trying to prevent further deconditioning. But I certainly don’t provide input thinking that I’m going to be able to magically reverse the damage that’s sort of already been done, if that makes sense. (Participant 9: Physiotherapist)

There was a sense that frailty isn’t always reversible and those with more severe frailty may not be able to improve in the hospital setting. Multiple staff members reported that patient motivation and engagement were critical in determining prognosis and those who were frail sometimes did not have the motivation to improve their frailty. Even if community-based resources were available to access, and the patient was referred, they chose not to access them. If severe frailty was considered not to be reversible, or the patient was assessed to lack motivation, the health professional’s treatment approach would shift from a restorative program to a focus on arranging adequate supports and care on discharge.

But it also depends on how open they are to suggestions and how motivated they are to actually implement the changes we recommend, because they might physically be able to reverse it. But if they’re not open to actually taking on the suggestions and motivated to change, nothing’s going to change. (Participant 11: Occupational Therapist)

Hospital-based health professionals generally felt that they had adequate resources to identify and manage (but not reverse) frailty in the inpatient setting. They perceived that primary health care and community organizations lacked resources. Furthermore, they felt that frailty could go undetected and unmanaged in the community, leading to poor health outcomes and recurrent hospital admissions.

I think the community is where these people are suffering and that is why they are here. (Participant 5: Nurse)

So by the time she got to us, she hadn’t eaten anything, she was 35 kilos. She was still mobile, but there was not enough support to keep her at home in the community, so she had to go to a nursing home now. Whereas with a few months of intervention, she may have returned to a point—or if it was picked up earlier, then she may have returned to a point where she could have reversed some of that frailty and gone back home. (Participant 11: Occupational Therapist)

Multiple participants reported a need for improved case management of older people in the community to prevent or address frailty. They felt the General Practitioner played an important role in helping frail older people; however, felt that General Practitioners were under-resourced. They were critical of the My Aged Care system (the Australian Government aged care services system) and lack of allied health and case management in the community as “there are a lot of very lonely people in the community who are not looking after themselves… there’s just no eyes on people in the community” (Participant 7: Social Worker).

Patients with family members who could perform a case management role for the patient were considered to be better off. Participant 13 (Medical Practitioner) stated that “I think those patients that have strong advocates in their families will ultimately get what they need, but some that don’t or people don’t advocate enough, they kind of slip through the cracks.”

Health professionals demonstrated care and compassion for patients they looked after in the inpatient ward and a concern about their welfare when they were discharged from the hospital.

When you are discharging people to the community you say to yourself, you’ll be back in 7 days. Because there’s not enough out there to support people in the community. It’s quite sad, how you have to send them home sometimes. (Participant 7: Social Worker)

Health professionals were unsure about the usefulness of their handover to the community, and whether their discharge summaries to general practitioners and community services were read or used. They reported rarely getting any feedback or follow-up after a patient had been discharged.

### Similarities and Differences Between Sites and Disciplines

The domains and themes above were mostly consistent for both Southern Adelaide and Northern Sydney participants; however, there were some small differences identified between the two states. Southern Adelaide participants commonly reported that they would identify frailty on appearance or history, whereas no participants in Northern Sydney reported this. They were also inclined to talk about frailty as a judgment or label, while the Sydney health professionals were more likely to talk about it as a description.

I think it’ll depend on the patient, how well a label like that is received. (Participant 9: Physiotherapist—Adelaide)

I find the word frail quite interesting as well, because I think people typically think of it as an adjective. Like it’s a describing word, rather than being a diagnosis itself. So, I just think it’s quite interesting. (Participant 22: Physiotherapist—Sydney)

There seemed to be greater awareness of frailty in the Northern Sydney health professionals, which may partially explain small differences identified between the groups. Although five Sydney health professionals reported they had discussed frailty with a patient, no health professionals in Southern Adelaide reported they had done this. Three Sydney health professionals mentioned previous frailty research or interventions in Northern Sydney Local Health District, and named local clinicians who had an interest in frailty. One reported that there was an increase in the use of the term “frailty” by clinicians over the last few years.

I use it as a general category of the patient population that we would see. Whether I think someone is—whether they’re pre-frail or frail, I suppose they are terminology that is being used a lot more within the hospital setting, and within the physio department, because of the studies that have happened here that we hear about. Whether we’re using the terms exactly correctly, I don’t know. But, yeah, definitely a lot more in the last five years. (Participant 24: Physiotherapist—Sydney)

Sydney health professionals also appeared more likely to report that diagnosing frailty was helpful.

Overall, the level of knowledge and perceptions of frailty were comparable across the different health professional groups, with no significant differences identified.

### Diverse Cases and Minor Themes

Although the theme of frailty-associated stigma was strong in the vast majority of interviews, one health professional reported that they didn’t believe in any negative connotations or stigma associated with the word “frail.” They did, however, report talking about frailty with patients in a “round-about” way and not using the word “frail” with patients, which may indicate that they do see barriers to using the word even if it isn’t due to stigma. Another felt that “most” patients would be open to discussing frailty and didn’t report the same hesitancy about discussing frailty with patients as the majority of health professionals interviewed. The participant didn’t report any experience talking about frailty with patients; however, and instead reported talking about “falls risk” with patients they thought were frail.

Two minor themes that have not been covered in the above results were also identified. One was around frailty being an inevitable part of aging, and that older people can view frailty as a normal age-related change. This can make discussion about frailty easier, but also make it hard to motivate patients to make changes to address frailty.

I guess, from what I’ve seen, usually patients would probably know that they’re frail, but wouldn’t be aware that maybe there’s something that they can do to improve it. So, if you mention being frail, they would agree, but then either aren’t keen or aren’t aware of ways they could possibly improve that. They just think they’re frail and that’s it. (Participant 21: Physiotherapist)

Another subtheme that was mentioned by three health professionals was about the impact the COVID-19 pandemic had on older people with frailty. Participants mentioned that community-allied health treatment was less accessible, and that older people lost social and community activities and were forced to stay home which may have worsened their frailty.

## Discussion and Implications

This study explored the knowledge, beliefs, and attitudes about frailty held by health professionals in hospitals and specifically their perceptions and knowledge of frailty; communication practices on the topic of frailty; and self-described clinical management of patients with or suspected of frailty.

Overall, we found that the health professionals included in this study did not routinely screen for frailty and many felt that frailty screening wouldn’t add value to their patient management. Many health professionals reported being able to identify patients with frailty through a combination of appearance, function, and history. The ability to identify frailty through subjective means is in keeping with research in other stakeholder groups including older persons, general practitioners, and surgeons (Ambagtsheer et al., 2019, Archibald et al., 2020, 2021; Korenvain et al., 2018). Although there is conflicting evidence as to how accurate and useful clinical judgment is in identifying frailty, it is the most common approach in clinical settings for identifying frailty in our study and others (Ahmed et al., 2016; Hii et al., 2015; O’Neill et al., 2016; van Dam et al., 2022). It is, however, strongly recommended to use a validated measurement tool to identify frailty in the Asia-Pacific clinician practice guidelines. Superficial visual inspection “is likely to only identify severe frailty” and there are large variations in clinical judgment across specialties as well as heterogeneity in frailty presentation (Dent et al., 2017).

Participants reported that the term “frailty” is a negative and stigmatized term amongst older persons and they therefore choose not to use it. This perception aligns with previous findings on frailty perceptions held by older Australian adults (Archibald et al., 2021), and in another international frailty study (Warmoth et al., 2015). Older adults associate frailty with negative old-age stereotypes and associate frailty with disablement (Warmoth et al., 2015). When frailty is discussed with patients, it is done without mentioning the term “frail” and replacing it with other words, the most frequently mentioned in this study was “deconditioned.” Frailty is sometimes discussed with family members, usually to assist with explaining recommendations for discharge planning. This finding is consistent with another study of surgical healthcare practitioners who reported that they were more likely to use the term “frail” with colleagues and patients’ families and less likely to use it with patients (Archibald et al., 2020). Participants felt that using a term, such as deconditioning, which sounded to be more of a reversible health condition offered more optimism for the patients and was better received. Frailty and deconditioning are, however, fundamentally different conditions and replacing the word “frail” with “deconditioned” may also be evidence of a lack of knowledge about what frailty is.

The multidisciplinary nature of inpatient care means that health professionals are more likely to focus on individual aspects of frailty that fit within their discipline rather than frailty itself and this prohibits a multicomponent, holistic approach to treating frailty as a clinical condition. This is in keeping with the finding that staff are not screening for, and diagnosing, frailty, and rather using discipline-specific screening tools and diagnosing malnutrition or physical impairment and not frailty itself. For frailty screening to be embedded in routine inpatient care, one or more disciplines would need to adopt frailty screening. This is more likely to happen if health professionals see benefits for patient management and if frailty screening and management are simple and time efficient.

Although participants felt they had the resources to manage frailty on their wards, they also spoke of time pressures and workload challenges, which could affect prioritizing interventions for frail older people in their clinical work. As there was little knowledge of screening tools or multicomponent frailty interventions it is unclear if the health professionals interviewed knew what resources they would need to identify and manage frailty systematically and effectively. They did, however, clearly identify a perceived lack of resources for people with frailty in the community. Assessing resource availability in each health service is an important component of addressing barriers to implementation of frailty management guidelines.

This study utilized a robust qualitative methodology underpinned by the Framework Method of analysis, thus demonstrating several strengths in a thorough and robust system to identify and synthesize important themes. To our knowledge, this study is the first to explore inpatient multidisciplinary health professionals’ perceptions of frailty screening, communication, and management. Limitations of this study include a potential for selection bias in the results as we relied on staff to volunteer to be interviewed and it is possible that staff who consented to interview were more confident in their knowledge, beliefs, and clinical management of frailty. There was also possible bias with the interviewers being employed within the health network. This can cause potential power differentials depending on the interviewers’ and interviewees’ roles within the organization, as well as create a risk of assumed understanding (Aburn et al., 2021). This study was conducted during the COVID-19 pandemic, whereby the impact of reduced workforce and hospital demand could have influenced participants’ perception of best-practice frailty management and delivery of hospital care.

The differences identified between health professionals in Southern Adelaide and Northern Sydney seem to reflect a greater exposure to frailty research and education in Northern Sydney, as well as an impact of well-known clinical champions of frailty diagnosis and intervention. Frailty appeared more likely to be discussed in Northern Sydney health professional clinical settings, and more likely to be used as a diagnosis or to describe a patient, as compared to Southern Adelaide health professionals. This is in keeping with evidence that a leader or champion can be effective in increasing implementation of interventions in organizations (Correa et al., 2020). Having clinical champions of frailty may increase frailty screening and management in acute settings.

Enablers to frailty guideline implementation identified in this study include the caring and compassionate attitudes displayed by the health professionals interviewed toward older people with frailty. They also displayed knowledge within their disciplines about management of individual components of frailty (e.g., dietetic approaches to weight loss). Barriers identified include lack of knowledge about frailty and a perceived stigma associated with the word “frail.” The multidisciplinary team approach contributes to individual health professionals focusing on their discipline-specific screening tools and management, and not taking ownership of a condition such as frailty, which bridges multiple disciplines. There was also a lack of a systematic approach to frailty identification and management as well as potential resource considerations both in the hospital and community.

For clinical practice to adopt clinical frailty guidelines, there is a need for greater uptake of frailty screening and improved management of frailty. The knowledge, attitudes, and experiences of health professionals are the potential barriers and/or enablers to the implementation of clinical guidelines. This study identifies the need for targeted education about frailty identification, reversibility, and management to improve frailty identification and management in hospitals. There is a need for staff to see value in frailty diagnosis and ownership about frailty as a clinical condition within their professional role in the multidisciplinary team. Health professionals would benefit from clear guidelines around which member of the team should be systematically diagnosing frailty and how to communicate this diagnosis and management within their team. Further research is needed to explore and identify communication techniques that help health professionals to reframe the topic of frailty from the personal (e.g., personal inadequacy) to the clinical while emphasizing frailty as a dynamic state (Archibald et al., 2021; Gill et al., 2006) when discussing frailty with patients. Targeted implementation strategies, such as local clinical champions of frailty, may help knowledge translation and implementation of clinical practice guideline recommendations for frailty screening and management in hospitals.

## Supplementary Material

gnae041_suppl_Supplementary_Materials

## Data Availability

Data are available on reasonable request. The data sets used and/or analyzed during this study will be available from the corresponding author on reasonable request after the publication of results. This study was not preregistered.
