# Supplementary material for: Hospital-Based Health Professionals’ Perceptions of Frailty in Older People
Source: Gerontologist. 2024 May 7;64(7):gnae041. doi: 10.1093/geront/gnae041 (PMC11181707; doi:10.1093/geront/gnae041)
Supplement: gnae041_suppl_Supplementary_Materials [file gnae041_suppl_supplementary_materials.docx]

Supplementary Material

Section A: Consolidated Criteria for Reporting Qualitative Research Guidelines

The reporting of the qualitative methods was checked against the COREQ (a 32-item checklist for interviews and focus groups).

| **Number** | **Item** | **Guide questions/descriptions** | **Cross-reference where included in report/reason for non-inclusion** |
| --- | --- | --- | --- |
| **Domain 1: research team and reflexivity** | | | |
| **Personal characteristics** | | | |
|  | Interviewer/facilitator | Which author conducted the interview or focus groups? | P.8. Data Collection. |
|  | Credentials | What were the researcher’s credentials? | P.8. Data Collection. |
|  | Occupation | What was their occupation at the time of the study? | P.8. Data Collection. |
|  | Gender | Was the researcher male or female? | P.8. Data Collection. |
| 1. ­ | Experience and training | What experience or training did the researcher have? | The researcher is a rehabilitation physician and is a current PhD candidate. She was supervised by Prof K La and guided throughout by the research team. |
| **Relationships with participants** | | | |
|  | Was a relationship established prior to study commencement? | Was a relationship established prior to study commencement? | P.8. Data collection. |
|  | What did the participants know about the researcher? | What did the participants know about the researcher? | P.8. Data collection. |
|  | Interviewer characteristics | What characteristics were reported about the interviewer/facilitator? | P.8. Data collection. |
| **Domain 2: study design** | | | |
| **Theoretical framework** | | | |
|  | Methodological orientation and theory | What methodological orientation was stated to underpin the study? e.g. grounded theory, discourse analysis, ethnography, phenomenology, content analysis | P.9. Data analysis. |
| **Participant Selection** | | | |
|  | Sampling | How were participants selected? e.g. purposive, convenience, consecutive, snowball | P.7. Participants and Setting |
|  | Method of approach | How were participants approached? e.g. face-to-face, telephone, mail, email | P.7. Participants and Setting |
|  | Sample Size | How many participants were in the study? | P.7. Participants and Setting |
|  | Non-participation | How many people refused to participate or dropped out? Reasons? | P.10. Results. |
| **Setting** | | | |
|  | Setting of data collection | Where was the data collected? e.g. home, clinic, workplace | P.10. Results. |
|  | Presence of non-participants | Was anyone else present besides the participants and researchers? | P.10. Results. |
|  | Description of sample | What are the important characteristics of the sample? e.g. demographic data, date | Table 2. |
| **Data collection** | | | |
|  | Interview guide | Were questions, prompts, guides provided by the authors? Was it pilot tested? | Supplementary Material, Section B. The interview guide was not pilot tested. |
|  | Repeat interviews | Were repeat interviews carried out? If yes, how many? | No. |
|  | Audio-visual recording | Did the research use audio or visual recording to collect the data? | P.8. Data Collection. |
|  | Field notes | Were ﬁeld notes made during and/or after the interview or focus group? | No. |
|  | Duration | What was the duration of the inter views or focus group? | P.8. Data Collection. |
|  | Data saturation | Was data saturation discussed? | P.8. Data Collection. |
|  | Transcripts returned | Were transcripts returned to participants for comment and/or correction? | No. |
| **Domain 3: analysis and findings** | | | |
| **Data analysis** | | | |
|  | Number of data coders | How many data coders coded the data? | P.9. Data analysis. |
|  | Description of the coding tree | Did authors provide a description of the coding tree? | P.10. Results. |
|  | Derivation of themes | Were themes identiﬁed in advance or derived from the data? | P.9. Data analysis. |
|  | Software | What software, if applicable, was used to manage the data? | P.9. Data analysis. |
|  | Participant checking | Did participants provide feedback on the ﬁndings? | No. |
| **Reporting** | | | |
|  | Quotations presented | Were participant quotations presented to illustrate the themes/ﬁndings? Was each quotation identiﬁed? e.g. participant number | P.10. Results. |
|  | Data and findings consistent | Was there consistency between the data presented and the ﬁndings? | Yes. |
|  | Clarity of major themes | Were major themes clearly presented in the ﬁndings? | P.10. Results. Table 3. |
|  | Clarity of minor themes | Is there a description of diverse cases or discussion of minor themes? | P.17. Results. |

Section B: Staff Frailty Perceptions and Knowledge Focus Group / Interview Guide

To be conducted with ward-based staff on the intervention wards prior to intervention and following intervention

1. Can you tell me a bit about the patient group that you work with?
2. What do you think of when you hear the term frailty? *(Prompts: what are some features or characteristics that you would associate with frailty?)*
3. Do you think frailty can be detected, delayed or reversed? *Prompts: do you think that once someone is frail, they are likely to always be frail?)* What has been your experience of seeing this in practice?
4. Do you think that patients want to know if they are Frail? Why / Why not? *(Prompts: Is there a stigma about frailty? Do patients have an emotional response to the diagnosis of frailty?)*
5. Is there any benefit to diagnosing someone with frailty? If so, what benefits do you foresee?
6. Have you ever discussed frailty with a patient? Do you think that it is a difficulty conversation to have? *(Prompts: If you haven’t personally, have you ever witnessed a colleague diagnosing and discussing frailty with a patient? How do patients respond?)*
7. Are frailty screening tools useful? Which ones have you used, and which do you prefer? *(if unable to name any, ask if they have heard of the FRAIL Scale, the Edmonton Frailty Scale, etc. Would they feel comfortable administering these assessments? Do they help you to diagnose frailty, or to discuss it with patients?*)
8. Are there enough resources in your current work setting to diagnose and address frailty?
9. What are the best ways to address frailty in the community?
10. How important is the translation of knowledge from hospital to the GP? – discuss phone calls / discharge summaries.
11. Do you have any other comments you’d like to make regarding any of the things we’ve discussed, or anything you think that is relevant that we haven’t discussed?

Thank you for your time and input.
